# Feeding *Limosilactobacillus fermentum* K9-2 and *Lacticaseibacillus casei* K9-1, or *Limosilactobacillus reuteri* TMW1.656 Reduces Pathogen Load in Weanling Pigs

**DOI:** 10.3389/fmicb.2020.608293

**Published:** 2020-12-17

**Authors:** Weilan Wang, Ruurd T. Zijlstra, Michael G. Gänzle

**Affiliations:** Department of Agricultural, Food and Nutritional Science, University of Alberta, Edmonton, AB, Canada

**Keywords:** probiotics, *Lactobacillus fermentum*, *Lactobacillus casei*, *Lactobacillus reuteri*, reutericyclin, ETEC, weanling pigs

## Abstract

Applying probiotics to improve gut health and growth performance of pigs is considered an effective approach to reduce use of antimicrobial growth promoters in swine production. Understanding the properties of these probiotics is a prerequisite for the selection of probiotic strains for pigs. Host-adapted probiotic strains were suggested to exert probiotic effects by different mechanisms when compared to free-living or nomadic probiotic strains. This study assessed the effect of probiotic intervention with *Limosilactobacillus reuteri* TMW1.656, a host-adapted species producing the antimicrobial compound reutericyclin, its isogenic and reutericyclin-negative *L. reuteri* TMW1.656Δ*rtcN*, and with *Limosilactobacillus fermentum* and *Lacticaseibacillus casei*, two species with a nomadic lifestyle. Probiotic strains were supplemented to the post weaning diet in piglets by fermented feed or as freeze-dried cultures. The composition of fecal microbiota was determined by high throughput sequencing of 16S rRNA gene sequence tags; Enterotoxigenic *Escherichia coli* and *Clostridium perfringens* were quantified by qPCR targeting specific virulence factors. Inclusion of host-adapted *L. reuteri* effectively reduced ETEC abundance in swine intestine. In contrast, nomadic *L. fermentum* and *L. casei* did not show inhibitory effects on ETEC but reduced the abundance of *Clostridium* spp. In addition, the increasing abundance of *Bacteriodetes* after weaning was correlated to a reduction of ETEC abundance. Remarkably, the early colonization of piglets with ETEC was impacted by maternal-neonatal transmission; the pattern of virulence factors changed significantly over time after weaning. Probiotic intervention or the production of reutericyclin showed limited effect on the overall composition of commensal gut microbiota. In conclusion, the present study provided evidence that the lifestyle of lactobacilli is a relevant criterion for selection of probiotic cultures while the production of antimicrobial compounds has only minor effects.

## Introduction

The stress triggered by weaning and the immature immune system contributes to the susceptibility of weanling pig to gut microbial dysbiosis and pathogen infection (Katsuda et al., 2006; Gresse et al., 2017). *Clostridium perfingens* Type C infections are common in neonatal piglets within 7 days of farrowing; infections with enterotoxigenic *Escherichia coli* (ETEC) are the most significant cause of post-weaning diarrhea (Kyriakis et al., 1996; Fairbrother et al., 2005). Vaccination against ETEC infections provides only limited protection because ETEC are non-invasive pathogens that reside in the intestinal lumen (Felder et al., 2001; Katrien et al., 2010). Alternatives for protection against pathogen infection are required particularly for early weaned pigs after the interrupted protection from maternal immunity. Probiotic bacteria are one of those alternatives and the primary trait required for the application of probiotics in weanling pigs is the ability to inhibit colonization of pathogen.

Probiotic bacteria have been shown to reduce the pathogen load, particularly *Salmonella enterica* and ETEC, in suckling and weaned piglets (Liao and Nyachoti, 2017; Wang and Gänzle, 2019). *Bacillus* spp. are most widely used as probiotics in swine production because their endospores remain viable throughout feed processing and storage at ambient temperature (Liao and Nyachoti, 2017; Ritter et al., 2018). Lactic acid bacteria and particularly lactobacilli have been used experimentally to reduce the pathogen load in suckling and weaned piglets (Liao and Nyachoti, 2017). Lactobacilli are freeze-dried to maintain their viability and functionality for use as probiotics (Iaconelli et al., 2015). In animal production, feed fermentation with lactic acid bacteria is also used; feed fermentation with probiotic lactic acid bacteria not only serves as delivery system for viable probiotic bacteria but also improves the availability of nutrients (Marko et al., 2014; Yang et al., 2015a). Probiotic effects of strains that were administered to swine by these two methods of delivery methods, however, have not been compared directly.

Lactobacilli include three genera that are adapted to vertebrate hosts, i.e., *Lactobacillus*, *Ligilactobacillus*, and *Limosilactobacillus* species (Duar et al., 2017b; Zheng et al., 2020); other lactobacilli, however, are ecologically diverse and are equipped for temporary persistence in multiple habitats including environmental niches and the intestinal tract of vertebrates (Duar et al., 2017b; Zheng et al., 2020). Host-adapted lactobacilli that are stable members of the intestinal microbiota of swine include *Limosilactobacillus reuteri* (previously *Lactobacillus reuteri*, Zheng et al., 2020) and *Lactobacillus amylovorus* (Leser et al., 2002; Duar et al., 2017a); nomadic organisms with documented use as probiotics in swine include *Lactiplantibacillus plantarum*, *Limosilactobacillus fermentum*, and *Lacticaseibacillus rhamnosus* (previously all assigned to the genus *Lactobacillus*, Wang and Gänzle, 2019; Zheng et al., 2020). Recent studies suggest that host-adapted and nomadic probiotics differ with respect to their survival in the intestinal tract, and with respect to mechanisms of probiotic activity (Maldonado-Gómez et al., 2016; Walter et al., 2018; Zhao et al., 2018; Wang and Gänzle, 2019). Probiotic feeding of the host adapted *L. reuteri* TMW1.656 resulted in significantly higher cell counts of the probiotic strain during intestinal transit when compared to the nomadic organisms *L. fermentum* K9-2 and *Lacticaseibacillus casei* K9-1 that were provided at the same viable cell counts (Zhao et al., 2018), suggesting improved survival. Vertebrate host adapted lactobacilli appear to induce immune tolerance (Lin et al., 2008; Livingston et al., 2010). In contrast, probiotic strains that are allochthonous to pigs are more likely to stimulate a pro-inflammatory immune response against pathogen infection (Wang et al., 2009; Li et al., 2012; Scharek-Tedin et al., 2013; Zhou et al., 2015; Upadhaya et al., 2017). A differential interaction of host-adapted and nomadic lactobacilli with the immune system, however, has not been sufficiently documented.

This study aimed to test the hypothesis that host-adapted and nomadic lactobacilli have different effects on pathogen load and gut microbiota in weanling pigs. Experiments employed commercially available strains of the nomadic species *L. fermentum* and *L. casei*, and the host adapted *L. reuteri*. *L. reuteri* TMW 1.656, which was previously shown to reduce the pathogen load in weanling pigs. Moreover, its ability to produce reutericyclin was suggested to modulate the development of fecal microbiota of weanling pigs (Yang et al., 2015b). The present experiment was designed to (1) verify a contribution of reutericyclin formation in probiotic function of *L. reuteri*; (2) compare the effects of freeze-drying and feed fermentation on probiotic effects of *L. fermentum* and *L. casei*, and (3) compare the beneficial effects of lactobacilli with different ecological origins in weanling pigs.

## Materials and Methods

### Microorganisms and Growth Conditions

Reutericyclin-producing *L. reuteri* TMW1.656 and isogenic reutericyclin-negative *L. reuteri* TMW1.656Δ*rtcN* (Lin et al., 2015) were routinely grown on anaerobically on modified MRS agar (Meroth et al., 2003) at 37°C. Two commercial probiotics *L. casei* K9-1 and *L. fermentum* K9-2 were provided by CanBiocin Inc. (Edmonton, AB, Canada) and grown under same conditions. Freeze-dried cultures of *L. casei* K9-1 and *L. fermentum* K9-2 with 10^9^ CFU/g vegetative cell were also provided by CanBiocin Inc. (Edmonton, AB, Canada), which were stored at 4°C until use.

### Experimental Diets and Animals

Overnight cultures of *L. reuteri* TMW1.656, *L. reuteri* TMW1.656Δ*rtcN*, *L. casei* K9-1, and *L. fermentum* K9-2 were applied for feed fermentation; a detailed characterization of the feed fermentation and the microbiological characteristics of the feed is provided by Zhao et al. (2018). Significant components of the dietary treatments are listed in Table 1. The fermentation was monitored by enumeration of viable cell counts, by measurement of the pH, and by quantification of the probiotic strains by strain-specific quantitative PCR (Zhao et al., 2018). Wheat was fermented with *L. reuteri* TMW1.656, *L. reuteri* TMW1.656Δ*rtcN*, or with *L. casei* and *L. fermentum*; the basal diet (98%) (Supplementary Table S1) was mixed with 2% fermented wheat. Control treatments included addition of unfermented, wheat that was incubated after chemical acidification, and unfermented wheat containing freeze dried cells of *L. casei* and *L. fermentum*. After addition of freeze-dried bacterial cells, the cell counts of *L. casei* and *L. fermentum* matched the cell counts of the same strains in fermented feed. The cell counts of *L. reuteri* TMW1.656, *L. reuteri* TMW1.656Δ*rtcN*, and *L. casei/L. fermentum* (freeze-fried and fermentation) in feed ranged from 7.7 ± 0.4 to 8.4 ± 0.5 log (cfu/g) (Zhao et al., 2018).

**TABLE 1:** Significant components of experimental diets used in this study.

Components	Control	Acidified control	*L. casei/L. fermentum*		*L. reuteri*	
			Freeze-dried	Fermentation	TMW 1.656	TMW 1.656 Δ*rtcN*
Acid (added or produced by fermentation)	−	+	−	+	+	+
*L. casei/L. fermentum*	−	−	+	+	−	−
*L. reuteri*	−	−	−	−	+	+
Reutericyclin	−	−	−	−	+	−

**+, Means the presence of component. −, Means the absence of component.**

The feeding trial was approved by the University of Alberta Animal Care and Use Committee for Livestock (Protocol # AUP00000182). A total of 48 male piglets (Duroc × Large White/Landrace F1) with similar bodyweight (6–7 kg) were selected at weaning (21 days of age). Six experimental diets were allocated to 48 piglets with a completely randomized block design, which provided 8 replicates per dietary treatment. All pigs were raised as previous described (Zhao et al., 2018) and weighted at the end of each week. Diets were provided twice per day with equal amounts. The content of dry matter (DM) in feed and leftover were measured each day to calculate the feed intake. The feed efficiency was calculated from average weight gain (g/day) over average feed intake (g DM/day).

### Samples Preparation and DNA Extraction

Feces were collected from the pen of each animal at days 0, 7, 14, and 21 and stored at −20°C immediately after sampling. Digesta samples were collected from ileum caecum and colon at euthanasia and stored at −20°C. Total bacterial DNA was extracted from 2 to 3 g of feces or digesta samples after thawing and mixing the frozen samples. Stool samples were homogenized with phosphate buffered saline (pH 7.4, Gibco, Thermo Fisher Scientific, Burlington, United States) and pre-treated by bead-beating (BioSpec Products, Inc., Bartlesville, United States) for 30 s × 8 times. Treated samples were heated at 95°C for 15 min to lyse cell before using QIAamp Fast DNA stool mini kit (Qiagen, Inc., Valencia, CA, United States). Only DNA samples with an A260/280 ratio higher than 1.8 were used for pathogen detection and microbial analysis. Purified DNA was diluted to 50 ng/μL for use.

### Detection of Intestinal Pathogens Using qPCR and HRM-qPCR

The detection of intestinal pathogens included species specific primers to enumerate *E. coli*, and primers targeting genes coding for fimbriae of swine-associated ETEC (F6, F18, F41, K88, and K99) as well as genes coding for toxins produced by ETEC (LT, Sta, and STb). The abundance of clostridial pathogens was determined by primers targeting the *Clostridium* cluster I and the gene coding for the α-toxin of *C. perfringens*. PCR was performed by multiplex HRM-qPCR and/or qPCR in 144 digesta and 191 fecal samples. Primers used in this study and their target genes are listed in Table 2. Genes coding for ETEC fimbriae genes were quantified by multiplex HRM-qPCR using Rotor-Gene Q (QIAGEN) HRM-thermo cycler and Type-it HRM Kit (QIAGEN) as described (Wang et al., 2017). A total volume of 25 μL HRM-qPCR reactions contained 12.5 μL 2 × HRM Master Mix, 3 μL template bacterial DNA, 200 nM × 5 targets primers. A standard curve for total content of five targets was established from 10-fold dilutions of the mixed standards, containing 2 × 10^2^ to 2 × 10^–8^ gene copies/μL of each purified positive control, which were amplified from stool samples with same conditions. The relative abundance of each target was quantified based on the linear correlation between the percentage of melting peak area and relative portion of the respective amplicon in mixed template (Wang et al., 2017). Total *E. coli*, ETEC toxin gens LT, Sta, and STb, *Clostridium* cluster I and *C. perfringens* were determined by qPCR using 7500 Fast real-time PCR system (Thermo Fisher Scientific, Burlington, United States). qPCR conditions were as previous described (Yang et al., 2015b; Wang et al., 2017).

**TABLE 2:** Primers used in this study.

Target gene	Sequence (5′→3′) (name)	Size (bp)	Tm (°C)
K88 fimbriae (*faeG*)	GCACATGCCTGGATGACTGGTG (K88 F)	439	63
	CGTCCGCAGAAGTAACCCCACCT (K88 R)		
K99 fimbriae (*fanA*)	CACTTGAGGGTATATGCGATCTT (K99 F)	92	62
	GACCTCAGTCACAGCAACTATAC (K99 R)		
F6 fimbriae (*fasA*)	GTTCCAGCCTCCAATGATACT (F6 F)	128	62
	GAAAGAGCTAATCCGCCATTTG (F6 R)		
F41 fimbriae Sub-unit A	GACCTCAGTCACAGCAACTATAC (F41 F)	110	62
	CGACCCGCAACATCCTTATT (F41 R)		
F18 fimbriae (*fedA*)	GGAGGTTAAGGCGTCGAATAG (F18 F)	90	62
	CCACCTTTCAGTTGAGCAGTA (F18 R)		
Universal stress protein A	CCGATACGCTGCCAATCAGT (UspA F)	884	66
	ACGCAGACCGTAGGCCAGAT (UspA R)		
*Clostridium* cluster I	GTGAAATGCGTAGAGATTAGGAA (CI F)	665	58
	GATYYGCGATTACTAGYAACTC (CI R)		
*C. perfringens* α-toxin	CTTGGAGAGGCTATGCACTATTT (CPα F)	90	60
	CTTAACATGTCCTGCGCTATCA (CPα R)		
Heat-labile enterotoxin	CCGTGCTGACTCTAGACCCCCA (LT F)	480	68
	CCTGCTAATCTGTAACCATCCTCTGC (LT R)		
Heat-stable enterotoxins	ATGAAAAAGCTAATGTTGGC (STa F)	193	65
	TACAACAAAGTTCACAGCAG (STa R)		
Heat-stable enterotoxins	TGCCTATGCATCTACACAAT (STb F)	113	60
	CTCCAGCAGTACCATCTCTA (STb R)		

### Fecal Microbiological Analysis Using 16S rRNA Gene Sequencing

A total of 191 samples of fecal community DNA was sequenced on pair end Illumina MiSeq platform (2 × 300 bp) by amplifying the V5-V6 domain of the 16S rRNA gene (University of Minnesota Genomics Center, Minneapolis, MN, United States). The forward and reverse primers sequences used for amplification were GTGCCAGCMGCCGCGGTAA and CGACRRCCATGCANCACCT, respectively. An average 17,440 paired 16S rRNA gene sequences per sample with an average length of 280 bp were retained after quality control and denoising from QIIME2 pipeline (QIIME2-2020.2) (Bolyen et al., 2019) by using “DADA2” (Callahan et al., 2016) with parameters: –p-trunc-len-f 277 –p-trunc-len-r 230.

Only sequence variants with relative abundance > 0.005% were retained for downstream analysis. Taxonomy was assigned to representative sequences by aligning to Silva release 132 classifier. Features classified as the genus *Lactobacillus* in QIIME2 were additionally blasted against the Genome Taxonomy Database release 95 (GTDB^1^) to reflect the current taxonomy of *Lactobacillaceae* (Zheng et al., 2020). Principle coordinates analysis (PCoA) and analysis of similarities (ANOSIM) were performed to explore microbial community differences through weighted UniFrac distance matrix. The sequence data are deposited at the National Center for Biotechnology Information (NCBI) under BioProject PRJNA665253.

### Statistical Analysis

The data for growth performance (average feed intake, average daily gain, and feed efficiency), intestinal pathogen load of *Clostridia* Cluster I, *C. perfringens* α toxin, *E. coli* and ETEC virulence factors were analyzed by linear mixed-effects model fitted for a completely randomized block design in R (version 5.12.6, The R Foundation for Statistical Computing, 2020). In this model, pig was considered the experimental unit; time, dietary treatments, and time × dietary treatments were calculated as fixed factors; the difference among blocks were regarded as random effects. The normality and homogeneity of all variables were determined by Shapiro-Wilk normality test and Bartlett test, respectively. The date for relative abundance of sequences variants and alpha diversity were analyzed by Kruskal-Wallis rank-sum test in R. The pairwise comparisons were performed with Wilcoxon rank sum test. Analysis of similarities (ANOSIM) for ETEC virulence factors data was calculated by Bray–Curtis dissimilarity; principle component analysis (PCA) for ETEC virulence factors data was analyzed using “kassambara/factoextra” packages in R. ANOSIM and Principle coordinate analysis (PCoA) for 16S rRNA sequence data was calculated from weighted UniFrac distance matrix. Results were presented as means ± standard deviation. *P* < 0.05 with Bonferroni-adjustment were considered significant.

## Results

### Animal Health and Growth Performance of Pigs

All pigs remained healthy and none of the pigs exhibited persistent diarrhea during the 3 weeks trial. The parameters reflecting growth performance are listed in Supplementary Table S1. The average feed intake and daily gain increased with time corresponding to feed efficiency increased from 0.65 to 0.8 kg during the first 2 weeks and remained stable in the third week (data not shown). No significant difference in feed efficiency was detected among dietary treatments or factors.

### Reduction of Pathogens and Toxins in Weanling Pigs by Feeding Probiotic Lactobacilli

**FIGURE 1:**
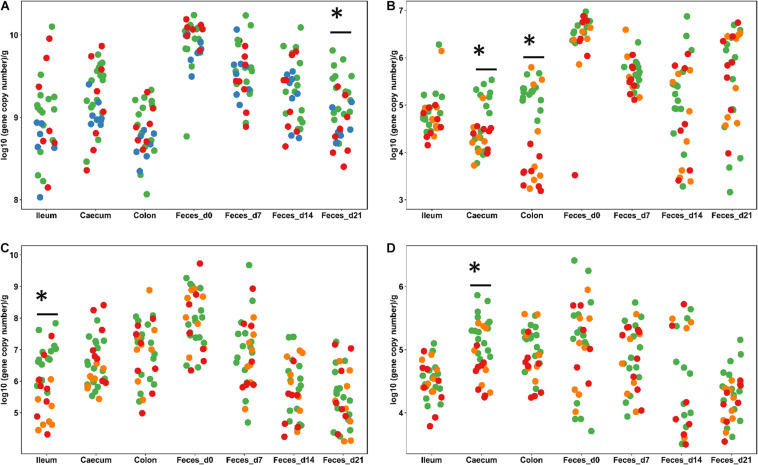
Abundance of *Clostridium* cluster I **(A)**, *C. perfringens* α-toxin **(B)**, *E. coli* **(C)** and the heat-labile toxin of ETEC **(D)** in weaned piglets. Digesta samples were collected from ileum, caecum, and colon at day 21. Feces was collected at day 0, 7, 14, and 21. All the specimens were analyzed by qPCR with specific primers. Each dot represents individual samples; samples are colored according to the dietary treatments. Only treatments with significant difference compared to the control are shown: Control and Acidified control (green, *n* = 16), freeze-dried *L. casei/L. fermentum* (blue, *n* = 8), fermented *L*. *casei/L. fermentum* (purple, *n* = 8), *L. reuteri* TMW1.656 (orange, *n* = 8) and *L. reuteri* TMW1.656ΔrtcN (red, *n* = 8). Data with asterisk (^∗^) are significantly different (*P* < 0.05) between dietary groups.

This study employed healthy animals that were housed under conditions that do not result in diarrhea; therefore, the abundance of genes coding for virulence factors was used as an indication of the ability of probiotics to prevent intestinal infections. The pathogen load in weanling pigs was determined by qPCR and HRM-qPCR. Primers targeted *Clostridium* Cluster I and the *C. perfringens* α-toxin and ETEC virulence factors including the heat labile toxin (LT), the heat stable toxin a (STa), the heat stable toxin b (STb), and K99, F18, F41, K88, and F6 fimbriae in weanling pigs. Fecal samples were obtained weekly and ileal, cecal, and colonic digesta were collected at day 21. The overall abundance of these pathogens and virulence factors were lower in the ileum than in the caecum and colon (Figure 1 and Supplementary Table S2) and decreased over time (Supplementary Table S3). The gene content of *Clostridium* Cluster I in digesta samples did not differ (*P* > 0.05) across dietary treatments (Figure 1 and Supplementary Table S2). Feeding freeze-dried *L. casei/L. fermentum* or *L. reuteri* TMW1.656Δ*rtcN* reduced (*P* < 0.05) *Clostridium* Cluster I in feces at day 21. Feeding *L. reuteri* TMW1.656 or *L. reuteri* TMW1.656Δ*rtcN* also decreased (*P* < 0.05) *C. perfringens* α toxin in the caecum and colon (Figure 1B and Supplementary Table S2).

Dietary intervention with probiotic lactobacilli had little influence on the abundance of *E. coli* and ETEC virulence factors. The abundance of *E. coli* decreased over time (Figure 1 and Supplementary Table S3); in ileal samples, the abundance of *E. coli* was reduced (*P* < 0.05) in samples from pigs fed with *L. reuteri* TMW1.656 or TMW1.656Δ*rtcN* (Figure 1C). The gene copy numbers of LT were also reduced (*P* < 0.05) in cecal samples from pigs fed with *L. reuteri* TMW1.656 or TMW1.656Δ*rtcN* (Figure 1C). The content of other ETEC virulence factors did not differ among diet groups but varied with aging.

### Longitudinal Changes of ETEC Virulence Factors in Swine Feces During the First 21 Days After Weaning

**FIGURE 2:**
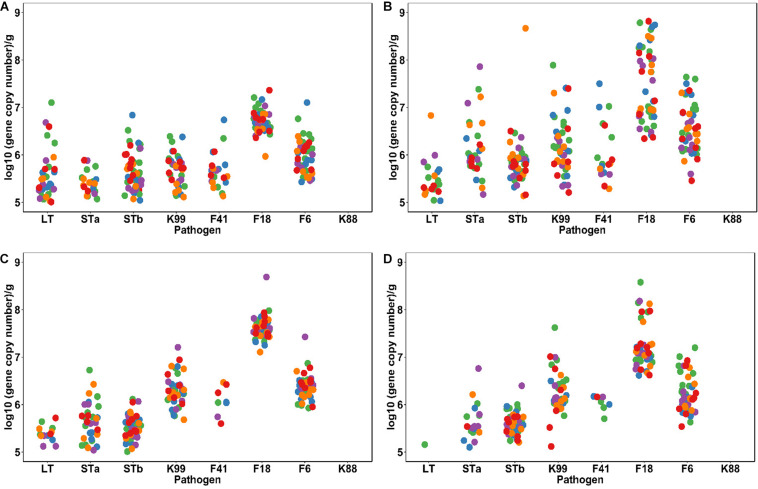
Dynamic profiles of ETEC virulence factors in fecal samples taken at weaning **(A)**, and 7 days **(B)**, 14 days **(C),** and 21 days **(D)** after weaning. All the samples were analyzed by qPCR with specific primers. Each dot represents individual fecal samples, colored according to the dietary treatments: Control and Acidified control (green, *n* = 16), freeze-dried *L. casei/L. fermentum* (blue, *n* = 8), fermented *L. casei/L. fermentum* (purple, *n* = 8), *L. reuteri* TMW1.656 (orange, *n* = 8) and *L. reuteri* TMW1.656 Δ*rtcN* (red, *n* = 8). Different diets did not influence (*P* > 0.05) the abundance of *E. coli* virulence factors.

The longitudinal profile of ETEC virulence factors throughout the 3 weeks after weaning is shown in Figure 2. The time after weaning strongly affected the abundance of *E. coli* and ETEC virulence factors. The load of these virulence factors was low at weaning and then displayed a chaotic profile during the first week followed by a steadily decrease during the following 2 weeks (Figure 2). LT decreased (*P* < 0.05) after the first week while STa were higher at day 7 (*P* < 0.05). F18 and F6 fimbriae increased (*P* < 0.05) and were the predominant ETEC fimbriae detected 7 days after weaning. Other fimbriae types and the STb toxin were detected in feces with low abundance (< 10^5^ copies/g) (Figure 2 and Supplementary Table S3); values were not different between dietary treatments or different time points.

**FIGURE 3:**
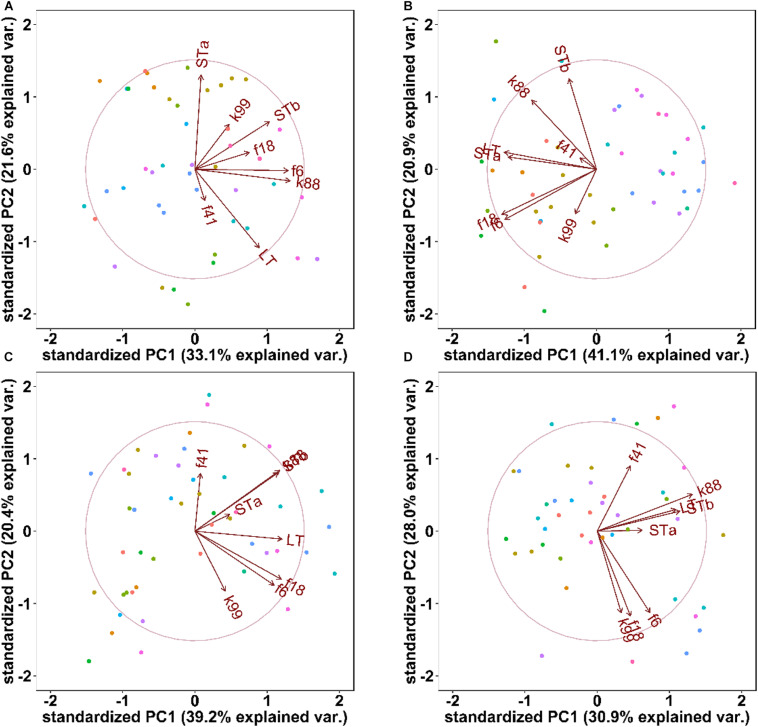
Principle component analysis of ETEC virulence factors at 0 days **(Panel A)**, 7 days **(B)**, 14 days **(C),** and 21 days **(D)** days after weaning. Each dot represents individual fecal samples, colored by sow: dots with same color are born from the same sow. The arrows represent the original variables, where the corresponding directions represent the correlations between the original variables and the principal components, and the length represents the contribution of the original data to the principal component.

Litter effects on the presence of virulence factors were evaluated by PCA (Figure 3) and by ANOSIM (Table 3). Seven days after weaning, PCA analysis of the abundance of ETEC virulence factors clustered pigs from different litters separately, this separation was not apparent 14 or 21 days after weaning (Figure 3). ANOSIM confirmed that sow or litter effects influenced (*P* < 0.05) the variations in ETEC virulence factors 7 days post-weaning but not at other time points; overall, the time after weaning significantly contributed to the variations through the experimental period (Table 3).

**TABLE 3:** ANOSIM^*a*^ of Bray–Curtis dissimilarity calculated with ETEC virulence factor genes content in feces at 0, 7, 14, and 21 days after weaning.

Time/factor	Time		Diet		Sow		Block	
	*R*^*b*^	*P-*value	*R*	*P-*value	*R*	*P*-value	*R*	*P-*value
Day 0	NA	NA	*NA*	NA	0.068	0.128	–0.046	0.954
Day 7	NA	NA	–0.033	0.909	0.403	0.001	–0.018	0.636
Day 14	NA	NA	–0.031	0.902	0.036	0.272	0.015	0.318
Day 21	NA	NA	0.035	0.111	0.0.42	0.247	–0.011	0.604
Overall	0.22	0.001	–0.006	0.817	–0.002	0.531	–0.001	0.537

**^*a*^ANOSIM, Analysis of similarity. ^*b*^An R-value near + 1 means that there is dissimilarity between the groups, while an R-value near 0 indicates no significant dissimilarity between the groups.**

### Composition of the Community of Lactobacilli and of the Overall Intestinal Microbiota After Feeding Lactobacilli to Weanling Pigs

**FIGURE 4:**
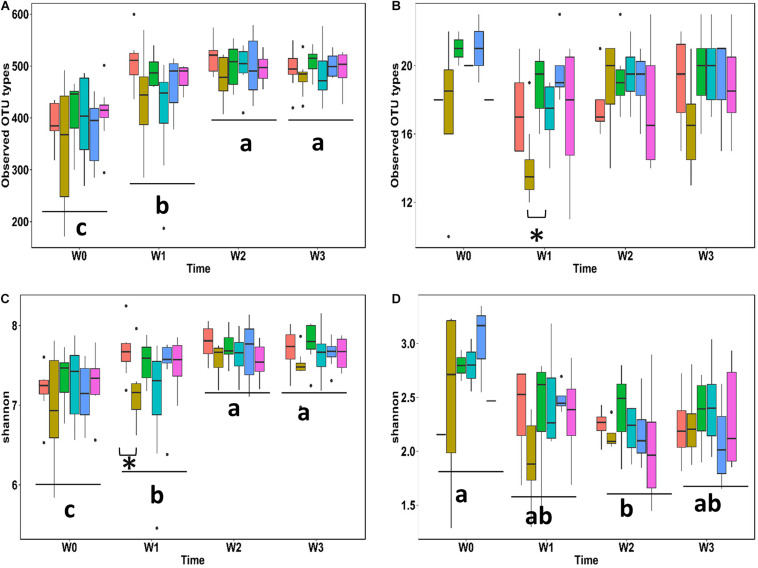
Fecal microbiota and diversity of lactobacilli during the first 3 weeks after weaning. Total number of bacteria **(A)** or lactobacilli **(B)** types observed and Shannon diversity index of fecal microbiota **(C)** or the community of lactobacilli **(D)** were analyzed based on partial 16S rRNA sequences. Each box represents one dietary treatment colored as following: Control (light red, *n* = 8), Acidified control (brown, *n* = 16), freeze-dried *L. casei/L. fermentum* (green, *n* = 8), fermented *L. casei/L. fermentum* (cyan, *n* = 8), *L. reuteri* TMW1.656 (light blue, *n* = 8) and *L. reuteri* TMW1.656 Δ*rtcN* (plum, *n* = 8). Indexes with different letters are significantly different (*P* < 0.05) between time points.

To determine the effects of probiotic lactobacilli on commensal microbes α-diversity (Figure 4) and β-diversity (Table 4 and Supplementary Figure S1) of fecal microbiota and *Lactobacillaceae* were assessed on the basis of 16S rRNA gene sequence data obtained with fecal samples from weanling pigs. The overall number of bacterial types in fecal microbiota significantly increased in the first 2 weeks and plateaued in the last week (Figure 4A); this was also reflected bacterial by an increased α-diversity as measured by the Shannon index (Figure 4C). Pigs feed with acidified wheat were noted for the reduction of α-diversity (Figure 4C, P < 0.05). The number of OTUs attributed to *Lactobacillaceae* remained overall stable while the diversity of the *Lactobacillaceae* community showed a transient decrease during the second week after weaning (Figure 4D). Dietary intervention with probiotic lactobacilli did not impact the diversity of the *Lactobacillaceae* community; in pigs fed with acidified wheat, the observed OTU types identified as intestinal lactobacilli was transiently reduced 7 days after weaning (*P* < 0.05; Figures 4B,D).

**TABLE 4:** ANOSIM^*a*^ of weighted UniFrac distance matrix calculated with partial 16S rRNA sequences.

		Fecal microbial		*Lactobacillaceae*	
Factor	Df	community		community	
		*R*^*b*^	*P*-value	*R*	*P-*value
Acid	1	–0.062	0.847	–0.013	0.609
Diet	5	0.008	0.125	0.025	0.028
Time of feeding	2	0.505	0.001	0.135	0.001
Litter	10	0.100	0.001	0.016	0.166
Host adapted vs. nomadic probiotics	2	–0.003	0.683	0.035	0.008

**^*a*^ANOSIM, Analysis of similarity. ^*b*^An R-value near + 1 means that there is dissimilarity between the groups, while an R-value near 0 indicates no significant dissimilarity between the groups.**

The dissimilarities between pigs fed with different lactobacilli were further assessed by analysis of β-diversity (Table 4 and Supplementary Figure S1). The overall fecal microbiota of weanling pigs was differentiated by the week of feeding and litters (*P* < 0.05, Table 4). Lactobacilli were also altered (*P* < 0.05) by the feed phase but not influenced by litter effects (*P* = 0.166). The inclusion of probiotic lactobacilli in feed did not change the structure of fecal microbiota (*P* = 0.683) but contributed to the difference of lactobacilli (*P* < 0.05). This influence was also reflected in an effect (*P* < 0.05) of dietary intervention on the composition of *Lactobacillaceae* communities.

### Contribution of Feeding Lactobacilli in Restoring Fecal Microbiota From Weaning Dysbiosis

**FIGURE 5:**
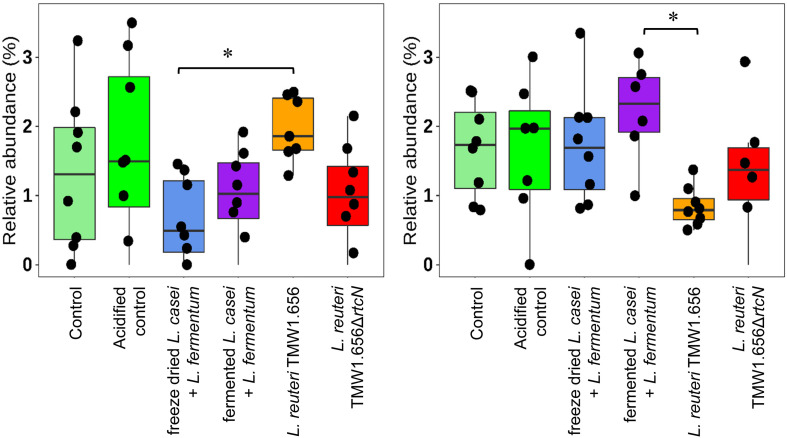
Relative abundance (%) of *Clostridium sensu strictu* **(left)** and *Limosilactobacillus* **(right)** in fecal microbiota of the pigs feed with experimental diets at 0 and 21 days after weaning, respectively. Data were calculated from sequencing of 16S rRNA tags using DADA2 workflow. Experimental diets were distinguished by color codes as shown: Control (light green, *n* = 8), acidified control (green, *n* = 8), freeze-dried *L. casei/L. fermentum* (blue, *n* = 8), fermented *L*. *casei/L. fermentum* (purple, *n* = 8), *L. reuteri* TMW1.656 (orange, *n* = 8) and *L. reuteri* TMW1.656ΔrtcN (red, *n* = 8). Data with asterisk (*) are significantly different (*P* < 0.05) between dietary groups.

The relative abundance of major bacterial taxa was compared to determine the effects of probiotic lactobacilli on the evolution of intestinal microbiota after weaning (Table 5 and Figure 5). Increased abundance was mainly detected in genus *Prevotella* and family *Prevotellaceae* from the phyla *Bacteroidetes*, genus *Lactobacillus, Limosilactobacillus, Agathobacter, Terrisporobacter*, and family *Ruminococcaceae* from the phyla *Firmicutes*. Reductions were mainly observed in *Bacteroidetes* classified of the genera *Bacteroides* and *Parabacteroides, Firmicutes* classified as *Christensenellaceae*, *Ruminococcaceae, Erysipelotrichaceae, Clostridiales, Lachnoclostridium* and most genera of the phylum *Proteobacteria*. Compared to control or acidified control diet, feeding lactobacilli did not change the relative abundance of fecal microbiota after weaning. Differences were only detected between pigs feeding with different lactobacilli. Feeding with *L. reuteri* TMW1.656 significantly increased relative abundance of *Clostridium sensu strictu* (*P* < 0.05) at day 0 compared to freeze-dried *L. fermentum* and *L. casei* but decreased *Limosilactobacillus* (previously known as *L. reuteri* group) compared to *L. fermentum* and *L. casei* at day 21.

**TABLE 5:** Bacterial genera with significant difference in fecal microbiota of pigs during the first 3 weeks after weaning, determined by sequencing of 16S rRNA tags.

Genus	Day 0	Day 7	Day 14	Day 21
***Actinobacteria***				
*Bifidobacterium*	0.07 ± 0.03^*a*^	ND^*b*^	ND^*b*^	0.02 ± 0.02^*ab*^
*Olsenella*	0.05 ± 0.03^*b*^	0.16 ± 0.04^*ab*^	0.1 ± 0.04^*ab*^	0.28 ± 0.06^*a*^
*[F:Atopobiaceae]*	0.08 ± 0.02^*b*^	0.18 ± 0.04^*ab*^	0.12 ± 0.02^*ab*^	0.22 ± 0.03^*a*^
*Collinsella*	0.41 ± 0.1^*ab*^	0.57 ± 0.14^*a*^	0.09 ± 0.03^*b*^	0.13 ± 0.03^*ab*^
***Bacteroidetes***				
*Bacteroides*	2.31 ± 0.45^*a*^	0.35 ± 0.24^*b*^	0.01 ± 0.01^*b*^	0.01 ± 0.01^*b*^
*Butyricimonas*	0.39 ± 0.09^*a*^	0.03 ± 0.01^*b*^	ND^*b*^	ND^*b*^
*Alloprevotella*	0.16 ± 0.05^*b*^	0.06 ± 0.02^*c*^	0.26 ± 0.06^*ab*^	0.35 ± 0.06^*a*^
*Prevotella*	1.92 ± 0.36^*b*^	2.34 ± 0.32^*b*^	4.48 ± 0.56^*a*^	6.23 ± 0.58^*a*^
*[F:Prevotellaceae]*	4.74 ± 0.51^*c*^	4.99 ± 0.38^*bc*^	6.18 ± 0.35^*ab*^	6.89 ± 0.4^*a*^
*Alistipes*	0.13 ± 0.03^*a*^	0.02 ± 0.02^*b*^	ND^*b*^	ND^*b*^
*[F:Rikenellaceae]*	6.08 ± 0.67^*ab*^	3.44 ± 0.33^*b*^	5.65 ± 0.36^*a*^	4.86 ± 0.36^*a*^
*Parabacteroides*	1.43 ± 0.16^*a*^	0.43 ± 0.11^*b*^	0.38 ± 0.06^*b*^	0.28 ± 0.07^*b*^
***Firmicutes***				
*Enterococcus*	0.05 ± 0.03^*ab*^	0.51 ± 0.23^*a*^	ND^*b*^	0.06 ± 0.03^*ab*^
*Lactobacillus*	3.23 ± 0.51^*b*^	8.84 ± 1.07^*a*^	6.21 ± 0.62^*a*^	7.05 ± 0.52^*a*^
*Limosilactobacillus*	0.93 ± 0.17^*b*^	2.1 ± 0.28^*a*^	2.13 ± 0.22^*a*^	1.86 ± 0.17^*a*^
*Ligilactobacillus*	0.05 ± 0.02^*ab*^	0.19 ± 0.12^*a*^	0.01 ± 0.01^*ab*^	ND^*b*^
*Streptococcus*	0.31 ± 0.08^*a*^	0.08 ± 0.03^*b*^	0.06 ± 0.04^*b*^	0.13 ± 0.05^*b*^
*[F:Christensenellaceae]*	5.65 ± 0.38^*a*^	3.36 ± 0.36^*b*^	2.05 ± 0.21^*bc*^	1.4 ± 0.15^*c*^
*Clostridium sensu*	1.26 ± 0.14^*a*^	1.18 ± 0.26^*ab*^	0.82 ± 0.28^*b*^	1.18 ± 0.18^*a*^
*[O:Clostridiales]*	1.54 ± 0.15^*a*^	2.23 ± 0.27^*a*^	1.59 ± 0.12^*a*^	1.07 ± 0.1^*b*^
*Agathobacter*	0.02 ± 0.02^*b*^	1.02 ± 0.26^*a*^	0.83 ± 0.15^*a*^	1.17 ± 0.16^*a*^
*Anaerostipes*	ND^*c*^	0.39 ± 0.09^*b*^	0.84 ± 0.14^*a*^	0.5 ± 0.09^*ab*^
*Blautia*	0.18 ± 0.05^*b*^	0.44 ± 0.15^*ab*^	0.45 ± 0.09^*a*^	0.38 ± 0.08^*a*^
*Coprococcus*	0.39 ± 0.09^*b*^	1.09 ± 0.19^*a*^	0.59 ± 0.1^*ab*^	0.55 ± 0.06^*a*^
*Dorea*	ND^*b*^	0.28 ± 0.08^*a*^	0.18 ± 0.05^*a*^	0.14 ± 0.03^*a*^
*Eisenbergiella*	0.14 ± 0.03^*a*^	0.02 ± 0.01^*b*^	ND^*b*^	ND^*b*^
*Fusicatenibacter*	0.01 ± 0.01^*a*^	0.3 ± 0.08^*b*^	0.12 ± 0.03^*b*^	0.16 ± 0.03^*b*^
*Lachnoclostridium*	1.51 ± 0.15^*a*^	0.17 ± 0.05^*b*^	0.08 ± 0.03^*bc*^	0.01 ± 0.01^*c*^
*Pseudobutyrivibrio*	0.17 ± 0.04^*a*^	0.01 ± 0.01^*b*^	0.02 ± 0.01^*b*^	0.01 ± 0^*b*^
*Roseburia*	1.33 ± 0.26^*a*^	0.32 ± 0.09^*c*^	0.59 ± 0.11^*bc*^	1.06 ± 0.15^*ab*^
*Peptococcus*	0.09 ± 0.03^*c*^	0.1 ± 0.03^*bc*^	0.19 ± 0.03^*ab*^	0.27 ± 0.04^*a*^
*[F:Peptococcaceae]*	0.08 ± 0.02^*b*^	0.26 ± 0.05^*a*^	0.13 ± 0.03^*ab*^	0.06 ± 0.01^*b*^
*Romboutsia*	0.63 ± 0.08^*a*^	0.02 ± 0.01^*b*^	0.06 ± 0.05^*b*^	ND^*b*^
*Terrisporobacter*	0.51 ± 0.08^*ab*^	0.5 ± 0.13^*b*^	0.61 ± 0.14^*ab*^	1.18 ± 0.18^*a*^
*Faecalibacterium*	0.21 ± 0.07^*b*^	0.51 ± 0.1^*ab*^	0.71 ± 0.12^*a*^	0.76 ± 0.13^*a*^
*Ruminiclostridium*	0.95 ± 0.07^*a*^	0.6 ± 0.07^*b*^	0.51 ± 0.09^*bc*^	0.34 ± 0.05^*c*^
*[F:Ruminococcaceae]*	7.43 ± 0.37^*c*^	9.19 ± 0.48^*bc*^	11.82 ± 0.41^*a*^	10.01 ± 0.4^*b*^
*Ruminococcus*	0.86 ± 0.11^*a*^	0.39 ± 0.07^*b*^	0.45 ± 0.09^*b*^	0.53 ± 0.09^*ab*^
*Subdoligranulum*	0.76 ± 0.21^*b*^	1.25 ± 0.23^*a*^	0.73 ± 0.11^*ab*^	0.71 ± 0.09^*ab*^
*[F:Ruminococcaceae]*	8.84 ± 0.3^*a*^	7.46 ± 0.42^*b*^	7.43 ± 0.39^*b*^	6.37 ± 0.27^*b*^
*Catenisphaera*	0.36 ± 0.07^*a*^	0.15 ± 0.04^*ab*^	0.05 ± 0.02^*b*^	0.07 ± 0.02^*b*^
*Holdemanella*	0.74 ± 0.15^*a*^	0.31 ± 0.07^*b*^	0.24 ± 0.05^*b*^	0.2 ± 0.04^*b*^
*Sharpea*	0.12 ± 0.04^*a*^	0.01 ± 0.01^*b*^	ND^*b*^	ND^*b*^
*Solobacterium*	0.11 ± 0.04^*b*^	0.53 ± 0.07^*a*^	0.58 ± 0.06^*a*^	0.48 ± 0.06^*a*^
*Turicibacter*	0.41 ± 0.06^*a*^	0.28 ± 0.12^*b*^	0.12 ± 0.06^*b*^	0.1 ± 0.04^*b*^
*[F:Erysipelotrichaceae]*	2.02 ± 0.18^*a*^	0.71 ± 0.09^*b*^	0.75 ± 0.1^*b*^	0.8 ± 0.1^*b*^
*Acidaminococcus*	0.05 ± 0.03^*ab*^	ND^*b*^	0.09 ± 0.04^*ab*^	0.13 ± 0.03^*a*^
*Phascolarctobacterium*	3.14 ± 0.2^*a*^	1.25 ± 0.17^*b*^	1.17 ± 0.13^*b*^	1.11 ± 0.11^*b*^
*[F:Acidaminococcaceae]*	0.03 ± 0.02^*b*^	0.08 ± 0.03^*ab*^	0.22 ± 0.06^*a*^	0.12 ± 0.03^*ab*^
*Anaerovibrio*	0.59 ± 0.13^*b*^	0.6 ± 0.11^*ab*^	0.64 ± 0.1^*ab*^	0.95 ± 0.13^*a*^
*Megasphaera*	0.03 ± 0.02^*b*^	0.1 ± 0.05^*ab*^	0.05 ± 0.02^*ab*^	0.21 ± 0.05^*a*^
*Mitsuokella*	0.01 ± 0.01^*b*^	0.06 ± 0.04^*b*^	0.17 ± 0.06^*b*^	0.57 ± 0.12^*a*^
*[O:Selenomonadales]*	0.64 ± 0.1^*b*^	0.97 ± 0.2^*b*^	0.91 ± 0.16^*b*^	1.8 ± 0.21^*a*^
***Planctomycetes***				
*[F:Pirellulaceae]*	1.18 ± 0.18^*b*^	1.78 ± 0.27^*ab*^	2.22 ± 0.31^*a*^	1.71 ± 0.19^*ab*^
***Proteobacteria***				
*[O:Bradymonadales]*	0.08 ± 0.03^*b*^	0.09 ± 0.03^*b*^	0.26 ± 0.05^*a*^	0.22 ± 0.04^*a*^
*Desulfovibrio*	0.61 ± 0.1^*a*^	0.04 ± 0.01^*c*^	0.06 ± 0.02^*c*^	0.18 ± 0.03^*b*^
*Succinivibrio*	0.24 ± 0.12^*b*^	0.61 ± 0.15^*a*^	0.06 ± 0.02^*b*^	0.14 ± 0.04^*b*^
*Oxalobacter*	0.11 ± 0.02^*a*^	0.02 ± 0^*b*^	0.12 ± 0.02^*b*^	0.14 ± 0.02^*b*^
*Escherichia-Shigella*	0.72 ± 0.28	0.73 ± 0.29	0.04 ± 0.02	0.16 ± 0.05
*[F:Enterobacteriaceae]*	0.54 ± 0.11	0.21 ± 0.06	0.07 ± 0.05	0.22 ± 0.12
***Synergistetes***				
*Cloacibacillus*	1.55 ± 0.25^*a*^	0.29 ± 0.09^*b*^	0.03 ± 0.01^*c*^	ND^*d*^
***Verrucomicrobia***				
*Akkermansia*	0.31 ± 0.12^*a*^	0.49 ± 0.17^*a*^	0.66 ± 0.28^*ab*^	0.26 ± 0.2^*b*^
*Unassigned*	2.14 ± 0.32^*b*^	2.15 ± 0.34^*b*^	3.25 ± 0.37^*ab*^	4.21 ± 0.35^*a*^

**Data were analyzed by QIIME pipeline and are represented as mean ± SEM of relative abundance (%). Data in the same row that do not share a common superscript are different (P < 0.05). ND means not detected. Unassigned genera are presented with upper level of family (F) or order (O) in square brackets. “Unassigned” means a good hit to a poorly defined taxonomy sequence. “Other” means the assignment is ambiguous.**

## Discussion

### Probiotic Activity of Host Adapted and Nomadic Lactobacilli

Immunomodulation and competitive colonization are among the major mechanisms of probiotics to inhibit pathogen infection (Hill et al., 2014). The ecological origin of the probiotic strain was suggested to determine whether one or the other is more important (Wang et al., 2009; Scharek-Tedin et al., 2013; Sonia et al., 2014; Zheng et al., 2016; Upadhaya et al., 2017). This study observed a reduction of *E. coli* and ETEC toxins mainly in pigs supplied with host-adapted *L. reuteri*. Anti-ETEC effects were also reported in studies of applying host-adapted *L. amylovorus* (previously *L. sobrius*) and *L. reuteri* (Kim et al., 2007; Yang et al., 2015a; Roselli et al., 2018). In contrast, the nomadic organisms *L. fermentum* and *L. casei* decreased the abundance of *Clostridium* cluster I but not of *E. coli*. Similar inhibition of *C. perfringens* was observed in weaning piglets that received *L. plantarum* but not in animals that received *L. reuteri* (Takahashi et al., 2008; Yang et al., 2015b). A possible explanation for this divergent effect is that host-adapted *L. reuteri* eliminated ETEC colonization through competitive exclusion in the small intestine. The strain used in the present study represents a rodent-adapted lineage of *L. reuteri* (Zheng et al., 2016), however, rodent-lineage strains of *L. reuteri* colonize the swine intestine equally well as strains from swine-adapted lineages (Duar et al., 2017a). However, the current study compared only small numbers of strains and the prevention of ETEC-induced diarrhea in piglets, along with an enhanced pro-inflammatory response, was also observed with the nomadic *L. rhamnosus* GG (Zhang et al., 2010). Therefore, differential effects of host adapted and nomadic lactobacilli on intestinal microbial ecology and host immunity remain to be validated in future studies that employ a larger number of host adapted and nomadic strains, and determine their impact on host immunity (Wang and Gänzle, 2019). The performance of *L. fermentum* and *L. casei* was not influenced by the mode of delivery, i.e., freeze dried or feed fermentation. Appropriate drying processes facilitate the development of probiotic cultures with high viability and stable immunomodulatory properties (Isolauri et al., 1991; Iaconelli et al., 2015). Of note, the strain *L. reuteri* TMW1.656 was more effective against *E. coli* when administered in higher cell counts (Yang et al., 2015a). Feed fermentations are a suitable tool to deliver adequate and high numbers of probiotic cells and may reduce cost in animal production. In addition, feed fermentations take advantages of nutritional advantages of feed fermentations (Stanton et al., 2005; Jørgensen et al., 2010).

### Impact of Reutericyclin on Intestinal Microbiota

Probiotic lactobacilli exerted only limited effects on the overall composition of fecal microbiota of weanling pigs. Metagenomic analysis of the same animal experiment demonstrated that the initial composition of fecal microbial community varied among litters and was then reshaped by feed over time, which agreed with significance of the diet in modulating development of swine gut microbiota (Wang et al., 2019). Reutericyclin-producing *L. reuteri* TMW1.656 reduced *Limosilactobacillus* (present study) and transiently inhibited other lactobacilli (Zhao et al., 2018). Bacteriocin expression by commensal bacteria or probiotic lactobacilli was suggested to favor their persistence in the host intestine and to enhance their probiotic effects (Kommineni et al., 2015). However, the low prevalence of DNA sequences coding for bacteriocins in human colonic microbiota implies that bacteriocin production is less relevant in the colon when compared to other body sites, particularly the oral cavity and the vagina (Kommineni et al., 2015; Zheng et al., 2015; Liu et al., 2016). Only few studies report on the relative impact of bacteriocin-producing and bacteriocin non-producing probiotics on swine intestinal microbiota, and even fewer compare isogenic mutant strains to account for strain-specific probiotic effects. A bacteriocin producing strain of *L. salivarius* exerted a subtle but significant impact on mouse and pig gut microbiota (Riboulet-Bisson et al., 2012). Likewise, previous studies that compared the reutericyclin producing *L. reuteri* TM1.656 with a reutericyclin-negative wild type strain of *L. reuteri* reported significant but minor changes in the composition of colonic microbiota (Yang et al., 2015b). Reutericyclin is produced to active concentrations in cereal fermentations (Gänzle and Vogel, 2003) and thus likely is also produced in the mainly wheat-based swine diet (this study). The comparison of *L. reuteri* TMW1.656 with the isogenic reutericyclin-negative strain *L. reuteri* TMW1.656Δ*rtcN* confirmed that the antimicrobial compound has only a limited impact on swine intestinal microbiota. In particular, the use of reutericyclin producing probiotic strains had no impact on the abundance of *C. perfringens*, an organisms that is sensitive to reutericyclin activity *in vitro* (Hofstetter et al., 2013). A possible explanation for the failure of *L. reuteri* to inhibit *C. perfringens* may be the site of the intestinal tract where the respective organisms are active. *L. reuteri* colonizes the osophagus of swine while *C. perfringens* colonizes the large intestine (Leser et al., 2002; Walter, 2008).

### Impact of Probiotic Intervention and Other Factors on Virulence Factors of Enterotoxigenic *Escherichia coli*

The primary cause of post weaning diarrhea, ETEC, uses diverse virulence factors to trigger diarrhea. In particular multiple different fimbriae are alternatively used to mediate adhesion to the small intestinal mucosa, and production of one or more of three diarrheal toxins causes fluid loss and diarrhea (Francis, 2002). Immunization with recombinant vaccines (Lin et al., 2013; Pereira et al., 2015) or the prevention of infection by analogs of the fimbriae receptor glycans (Shoaf-Sweeney and Hutkins, 2008; Chen et al., 2014) requires accurate diagnosis of dominant virulence factors. Our high-resolution analysis of ETEC virulence factors described the evolution of virulence factors of ETEC at suckling and post-weaning stages. As the receptor for F18 fimbriae is absent in neonatal pigs, the relevance of ETEC with F18 fimbriae increases post-weaning (Nagy et al., 1997; Fairbrother et al., 2005), an effect that was also observed in the current study. The decreasing abundance of the LT and an increased abundance of Sta confirms the higher frequency of ETEC expressing the combination of Stb/F18 and LT/K88 in weanling pigs (Wilson and Francis, 1986; Osek, 1999; Frydendahl, 2002). Few ETEC strains additionally carry Stx2e, which are referred as hybrid Shiga toxin-producing *E. coli*/ETEC (STEC/ETEC) but was not included in our detection spectrum (Leonard et al., 2016).

The quantification of virulence factors of ETEC also demonstrated that piglets from the same sow harbored a more similar profile of virulence factors when analyzed shortly after weaning. This demonstrates that maternal-neonatal transmission is an important contributor to the early colonization of ETEC. The importance of strain-level transmission of commensal organisms has been demonstrated for mothers and their children (Yassour et al., 2018) but not yet for sows and piglets. In consequence, maintaining good sanitary conditions and gut health of the sows are key to successful ETEC prevention in swine production.

Time or age significantly contributed to the variations in patterns of ETEC virulence factors (this study) and microbiota assembly in weanling pigs (Wang et al., 2019). Therefore, it was not accidental that the longitudinal profiles of ETEC virulence factor coincided with development of gut microbiota in weanling pigs. In previous studies, the abundance of *Bacteroidetes*, including genera from *Prevotellaceae*, *Lachnospiraceae*, and *Ruminococcaceae* families were higher in health pig than that in diarrheal pigs (Gorkiewicz et al., 2013; Dou et al., 2017), which positively correlated with the increasing colonization of *Bacteroidetes* after weaning (Molist et al., 2012). Thus, promoting an earlier colonization of *Bacteroidetes* may be a strategy to decease the incidence of ETEC infection in early weaned pigs.

In conclusion, host-adapted and nomadic probiotic lactobacilli inhibited different pathogens in healthy weanling pigs. The host adapted *L. reuteri* was more effective against ETEC while nomadic *L. casei* and *L. fermentum* were effective against *C. perfringens*. Bacteriocin production was less relevant to antimicrobial effects of lactobacilli in the colon. Early colonization of ETEC was associated with maternal-neonatal transmission and decreased with colonization of *Bacteroidetes* over time after weaning. Feed fermentation with probiotic strains may find a wider application in animal production as an appropriate tool for cost-effective delivery of probiotic strains.

## Data Availability Statement

The sequence data are deposited at the National Center for Biotechnology Information (NCBI) under BioProject PRJNA665253.

## Ethics Statement

The animal study was reviewed and approved by the University of Alberta Animal Care and Use Committee for Livestock.

## Author Contributions

MG and RZ conceived and designed the experiments. WW performed the experiments and analyzed the data. WW and MG wrote the manuscript. WW, MG, and RZ reviewed the manuscript. All authors read and approved the final manuscript.

## Conflict of Interest

The authors declare that the research was conducted in the absence of any commercial or financial relationships that could be construed as a potential conflict of interest.

## Footnotes

1 https://gtdb.ecogenomic.org/
