# Supplementary material for: Feeding Limosilactobacillus fermentum K9-2 and Lacticaseibacillus casei K9-1, or Limosilactobacillus reuteri TMW1.656 Reduces Pathogen Load in Weanling Pigs
Source: Front Microbiol. 2020 Dec 17;11:608293. doi: 10.3389/fmicb.2020.608293 (PMC7773707; doi:10.3389/fmicb.2020.608293)

Online supplementary material to

Feeding *Limosilactobacillus fermentum*, *Lacticaseibacillus casei*, or *Limosilactobacillus reuteri* reduces pathogen load in weanling pigs

Weilan Wang, Ruurd T. Zijlstra, Michael G. Gänzle*

[1] Department of Agricultural, Food and Nutritional Science, University of Alberta, Edmonton, Alberta, Canada;

Table S1. Growth performance of pigs during the first 3 weeks after weaning

Table S2. Gene copy number for *Clostridium* Cluster I, *C. perfringens* α toxin I, LT, STa, STb, E. coli, K99, F41, F18, F6 and K88 fimbriae in digesta collected from ileum, caecum and colon at day 21 [a]

Table S3. Gene copy number for *Clostridium* Cluster I, *C. perfringens* α toxin I, LT, STa, STb, *E. coli*, K99, F41, F18, F6 and K88 fimbriae in feces collected on days 0, 7, 14, and 21 [a]

Figure S1. Fecal microbiota (left) and *Lactobacillaceae* (right) community structure as determined by Principle coordinates analysis (PCoA) of partial 16S rRNA sequences. Each dot represents individual fecal samples, colored according to the feed phase (left) and according to the wheat content of feed (right).

Table S1. Growth performance of pigs during the first 3 weeks after weaning

Dietary treatment	Average feed intake (g DM [a] /day)	Average daily gain (g / day)	Feed efficiency (G / F)
Control	499±20.4	406±72.3	0.81±0.13
Acidified control	485±25.3	343±78.1	0.70±0.14
L. casei / L. fermentum			
Freeze-dried	493±15.9	378±49.0	0.77±0.10
Fermented	514±83.9	376±86.9	0.74±0.19
L. reuteri			
TMW1.656	489±29.1	384±76.0	0.78±0.14
TMW1.656*ΔrtcN*	489±16.8	381±49.6	0.78±0.10
P value [b]			
Acid	0.61	0.11	0.08
L. casei / L. fermentum	0.82	0.90	0.63
L. reuteri	0.05	0.74	0.14
Fermentation	0.09	0.95	0.28
Reutericyclin	0.51	0.93	0.80

[a] DM, dry matter.

[b] For acid effects, *P* values are for the control diet versus the chemically-acidified diet; For fermentation, *P* values are for the freeze-dried *L. casei / L. fermentum*-containing diet versus fermented *L. casei / L. fermentum*-containing diet; For *L. casei / L. fermentum*, *P* values are for the comparisons between *L. casei / L. fermentum*-containing diets (freeze-dried and Fermented) and unfermented diets (Control and Acidified control); For *L. reuteri*, *P* values are for the *L. reuteri*-containing diets (TMW1.656 and TMW1.656*ΔrtcN*) versus unfermented diets (Control and Acidified control); For reutericyclin, *P* values are for the *L. reuteri*-containing diets (TMW1.656 and TMW1.656*ΔrtcN*)

Table S2. Gene copy number for *Clostridium* Cluster I, *C. perfringens* α toxin I, LT, STa, STb, E. coli, K99, F41, F18, F6 and K88 fimbriae in digesta collected from ileum, caecum and colon at day 21 [a]

Bacterium or bacterial toxin and segments	Log (gene copy no./g) for the following diet:					
	Control	Acidified control	*L. casei / L. fermentum* freeze-dried	*L. casei / L. fermentum*	*L. reuteri* TMW1.656	*L. reuteri* TMW 1.656 Δ*rtcN*
Clostridium Cluster I						
Ileum	9.01±0.23 ^{B}	8.81±0.97 $^{A, B}$	8.42±0.53 ^{B}	8.66±0.69 ^{B}	8.73±0.77	8.96±0.76
Caecum	9.38±0.23 ^{A}	9.26±0.46 ^{A}	9.04±0.17 ^{A}	9.16±0.54 ^{A}	9.04±0.40	9.17±0.55
Colon	8.97±0.40 ^{B}	8.68±0.49 ^{B}	8.57±0.28 ^{B}	8.75±0.41 $^{A, B}$	8.73±0.42	8.70±0.55
C. perfringens α toxin						
Ileum	4.71±0.18 ^{B}	5.08±0.56	4.79±0.25^{B}	4.79±0.36 ^{B}	4.85±0.56	4.65±0.31^{A}
Caecum	4.21±0.33 $^{Y, A}$	5.58±0.89 ^{X}	5.33±0.19 $^{X, A}$	5.38±0.32 $^{X, A}$	4.28±0.43 ^{Y}	4.30±0.25 $^{Y, A}$
Colon	5.34±0.15 $^{X, A}$	5.39±0.76 ^{X}	5.00±0.51 $^{X, Y, A, B}$	4.63±0.32 $^{XY, B}$	4.39±1.06 ^{Y}	< 4 $^{Z, B}$
LT						
Ileum	4.44±0.20 ^{C}	4.61±0.32 ^{C}	4.37±0.34 ^{B}	4.54±0.33 ^{C}	4.57±0.23 ^{B}	4.43±0.43
Caecum	5.07±0.18 $^{XYZ, B}$	5.53±0.20 $^{X, A}$	5.44±0.24 $^{XY, A}$	5.61±0.17 $^{X, A}$	4.91±0.43 $^{Y, A, B}$	4.61±0.29 ^{Z}
Colon	5.26±0.15 $^{X, A}$	4.93±0.32 $^{X, Y, B}$	5.10±0.43 $^{XY, A}$	5.13±0.28 $^{XY, B}$	5.02±0.40 $^{XY, A}$	4.62±0.36 ^{Y}
STa						
Ileum	< 4^{C}	< 4 ^{B}	< 4 ^{B}	< 4 ^{B}	< 4 ^{B}	< 4 ^{B}
Caecum	5.64±0.46 ^{A}	< 4 ^{A}	4.78±2.13 ^{A}	5.66±1.77 ^{A}	5.88±0.82 ^{B}	5.71±0.26 ^{A}
Colon	4.88±0.41 ^{B}	< 4 ^{B}	< 4 ^{B}	4.24±1.82 ^{A}	4.15±1.46 ^{B}	< 4^{B}
STb						
Ileum	4.04±0.41 ^{B}	4.12±0.20 ^{B}	< 4 ^{B}	< 4 ^{B}	4.25±0.40	4.05±0.43
Caecum	< 4 $^{Y, B}$	4.70±0.21 $^{X, A}$	4.80±0.82 $^{X, A}$	4.67±0.12 $^{X, A}$	< 4 ^{Y}	4.02±0.29 ^{XY}
Colon	4.51±0.28 ^{A}	4.13±0.50^{B}	4.63±0.80 ^{B}	4.45±0.38 ^{A}	4.24±0.66	< 4
E. coli						
Ileum	6.91±0.43 ^{X}	6.49±0.73 $^{X, Y}$	6.00±1.23 $^{X, Y}$	6.21±0.97 $^{X, Y}$	5.14±0.64 $^{Y, C}$	5.78±1.08 $^{XY, B}$
Caecum	6.77±0.56	6.36±0.84	5.63±1.51	6.02±0.85	6.04±0.35 $^{Y, B}$	7.09±0.93 ^{A}
Colon	7.33±0.67	6.58±0.95	5.66±1.23	5.74±0.69	7.06±1.05 ^{A}	6.65±1.02 ^{A}
K99 fimbriae						
Ileum	< 4 ^{B}	4.00±0.25 ^{B}	< 4	4.05±0.15 ^{B}	< 4 ^{B}	< 4 ^{B}
Caecum	4.91±0.19^{A}	4.89±0.24^{A}	4.32±0.57	4.61±0.20^{A}	4.67±0.31^{A}	4.56±0.27^{A}
Colon	< 4 ^{B}	< 4 ^{B}	4.01±0.44	4.07±0.12 ^{B}	< 4 ^{B}	< 4 ^{B}
F18 fimbriae						
Ileum	< 4 ^{B}	4.15±0.27 ^{B}	4.16±0.33	4.03±0.25	< 4 ^{B}	4.08±0.21 $^{A, B}$
Caecum	4.86±0.20 ^{A}	4.74±0.28 ^{A}	4.20±0.65	< 4	4.46±0.79 ^{A}	4.33±0.39 ^{A}
Colon	< 4 ^{B}	4.09±0.40 ^{B}	4.11±0.35	4.03±0.19	< 4 ^{B}	< 4 ^{B}
F6 fimbriae						
Ileum	< 4 ^{C}	4.19±0.17 ^{B}	4.06±0.22	< 4 ^{B}	4.09±0.16 ^{B}	4.25±0.17 ^{A}
Caecum	5.10±0.16 ^{A}	5.12±0.24 ^{A}	4.34±0.56 ^{Z}	4.65±0.26 ^{A}	4.90±0.36 ^{A}	4.45±0.50 ^{A}
Colon	4.07±0.30 ^{B}	4.27±0.25^{B}	< 4	4.05±0.12 ^{B}	< 4^{C}	< 4 ^{B}

[a] Data are presented means ± SD (n=48). Superscripts X and Y denote significant differences ($P < 0.05$) among diets at each segment (comparison across rows); superscripts A, B, C and D denote significant differences ($P < 0.05$) within a diet among segments (comparison across columns). Values that do not share a superscript are significantly different. The detection limit for *Clostridium Cluster* I*, C. perfringens* α toxin I, LT, STa, STb, *E. coli*, K99, F41, F18, F6 and K88 fimbriae was 4 log10 gene copies/g of feces (wet weight).

Table S3. Gene copy number for *Clostridium* Cluster I, *C. perfringens* α toxin I, LT, STa, STb, *E. coli*, K99, F41, F18, F6 and K88 fimbriae in feces collected on days 0, 7, 14, and 21 [a]

Bacterium or bacterial toxin and time (day)	Log (gene copy no./g) for the following diet:					
	Control	Acidified control	*L. casei / L. fermentum* freeze-dried	*L. casei / L. fermentum*	*L. reuteri* TMW1.656	*L. reuteri* TMW 1.656 Δ*rtcN*
***Clostridium Cluster* I**						
0	10.08±0.18^{A}	9.80±0.45^{A}	9.82±0.17^{A}	9.86±0.23^{A}	9.86±0.26^{A}	10.07±0.13^{A}
7	9.68±0.22^{B}	9.67±0.51$^{A, B}$	9.54±0.29^{B}	9.28±0.57^{B}	9.54±0.47^{B}	9.43±0.33$^{A, B}$
14	9.51±0.45$^{B, C}$	9.35±0.49$^{A, B}$	9.25±0.32^{C}	9.47±0.49$^{A, B}$	9.34±0.25$^{B, C}$	9.24±0.44$^{A, B}$
21	9.32±0.24$^{X, C}$	9.27±0.37$^{X, B}$	8.87±0.18$^{Y, D}$	9.06±0.47$^{X, Y, B}$	9.08±0.23$^{X, Y, C}$	8.80±0.32$^{Y, B}$
***C. perfringens* α toxin**						
0	6.84±0.40^{A}	7.05±0.55^{A,}	6.49±0.35^{A,}	6.65±0.70^{A}	6.71±0.65^{A}	6.26±1.18^{Y}
7	5.96±0.68$^{A, B}$	6.49±1.51$^{A, B}$	5.96±0.92^{A}	6.48±1.47^{A}	6.27±2.34^{A}	5.55±0.32
14	5.13±0.90^{B}	5.00±1.27^{C}	5.34±1.01^{B}	4.59±1.01^{B}	4.56±1.01^{B}	4.59±1.20
21	5.36±1.20^{B}	5.66±1.24$^{B, C}$	5.57±1.29$^{A, B}$	5.19±1.30^{B}	5.73±0.89$^{A, B}$	5.61±0.92
**LT**						
0	5.63±0.70^{A}	4.81±1.03	5.19±0.53^{A}	5.26±0.80^{A}	5.07±0.67^{A}	5.22±0.77
7	4.98±0.45^{A}	4.90±0.63	4.62±0.78$^{A, B}$	4.71±0.88$^{A, B}$	5.05±0.89^{A}	4.88±0.50
14	4.04±0.91^{B}	4.23±0.91	3.85±0.93^{C}	4.06±1.02^{B}	4.14±1.06^{B}	4.14±0.94
21	4.20±0.69^{B}	4.36±0.34	4.10±0.28$^{B, C}$	4.14±0.40^{B}	4.10±0.29^{B}	4.22±0.33
**STa**						
0	4.65±0.83	4.85±0.79	4.48±0.72	4.64±0.88	5.07±0.51$^{A, B}$	4.72±0.85
7	5.37±1.26	5.17±1.58	4.61±1.47	5.43±1.63	6.13±1.95^{A}	4.93±1.34
14	4.82±1.45	4.90±1.21	4.99±0.99	5.14±1.00	5.08±1.22$^{A, B}$	4.80±1.23
21	4.37±0.98	4.18±1.07	4.50±1.05	5.18±1.12	3.95±1.09^{C}	4.01±1.06
**STb**						
0	5.70±0.54	5.64±0.41^{A}	5.65±0.60	5.47±0.40	5.47±0.34^{B}	5.84±0.25
7	5.78±0.23	5.91±0.27^{A}	5.68±0.21	5.80±0.33	6.16±1.07^{A}	5.76±0.38
14	5.48±0.33	5.31±0.36^{B}	5.50±0.25	5.54±0.30	5.52±0.28^{B}	5.59±0.24
21	5.67±0.26	5.65±0.15^{A}	5.58±0.24	5.69±0.31	5.49±0.21^{B}	5.49±0.32
E. coli						
0	7.69±0.74^{A}	8.09±0.98^{A}	7.91±0.76^{A}	7.79±0.67^{A}	8.01±0.87^{A}	7.89±1.19^{A}
7	6.89±1.30^{A}	6.99±1.20^{B}	6.48±0.77^{B}	6.70±0.96^{B}	6.72±0.91^{B}	6.60±1.55^{B}
14	5.74±1.03^{B}	5.79±0.93^{C}	5.87±1.42$^{B, C}$	5.87±0.94^{C}	5.98±0.93$^{B, C}$	5.07±0.94^{C}
21	5.75±0.99^{B}	5.31±0.72^{C}	5.38±0.86^{C}	5.62±0.5^{C}	5.18±0.95^{C}	5.66±1.24$^{B, C}$
**K99 fimbriae**						
0	4.88±1.22^{B}	5.38±1.12	5.14±1.27^{B}	4.97±1.08^{B}	5.16±0.68^{B}	5.45±0.90
7	6.46±0.65^{A}	5.83±1.05	6.34±0.71^{A}	5.64±0.82^{B}	6.16±0.5^{A}	5.32±1.54
14	5.85±1.15^{A}	6.23±0.26	5.87±1.08^{A}	6.36±0.39^{A}	6.30±0.36^{A}	5.65±1.43
21	6.26±0.24^{A}	6.11±1.22	4.77±1.54^{B}	5.93±1.00^{A}	5.77±0.98$^{A, B}$	5.45±1.52
**F41 fimbriae**						
0	<4	<4	5.24±1.28	4.46±1.27	4.85±1.02	4.61±1.21
7	4.45±1.57	4.12±1.46	4.87±1.83	<4	4.15±1.25	4.57±1.48
14	<4	<4	<4	<4	<4	4.34±1.49
21	<4	4.06±1.13	<4	<4	<4	<4
**F18 fimbriae**						
0	6.74±0.16^{B}	6.77±0.28^{B}	6.72±0.21^{B}	6.72±0.21^{B}	6.57±0.31^{B}	6.74±0.31
7	7.52±0.80^{A}	7.67±0.97^{A}	7.73±0.91^{A}	7.27±0.66$^{A, B}$	7.55±0.69^{A}	7.44±0.90
14	7.49±0.14^{A}	7.61±0.17^{A}	7.56±0.21^{A}	7.75±0.40^{A}	7.54±0.22^{A}	7.66±0.18
21	7.12±0.31$^{A, B}$	7.48±0.64^{A}	6.98±0.22^{B}	7.16±0.44^{B}	7.18±0.49$^{A, B}$	7.35±0.46
**F6 fimbriae**						
0	6.08±0.28^{B}	6.02±0.44^{B}	5.97±0.52^{B}	5.79±0.30^{C}	5.87±0.37^{B}	6.02±0.23^{B}
7	6.61±0.51^{A}	6.67±0.63^{A}	6.70±0.60^{A}	6.34±0.42$^{A, B}$	6.54±0.42^{A}	6.41±0.59$^{A, B}$
14	6.24±0.20^{B}	6.40±0.25$^{A, B}$	6.30±0.17$^{A, B}$	6.55±0.36^{A}	6.30±0.21^{A}	6.45±0.24^{A}
21	6.26±0.35$^{A, B}$	6.51±0.45^{A}	5.98±0.22$^{, B}$	6.16±0.30$^{, B}$	6.24±0.45$^{A, B}$	6.28±0.43$^{A, B}$

[a] Data are presented means ± SD (n=48). Superscripts X and Y denote significant differences (P < 0.05) among diets at each time point (comparison across rows); superscripts A, B, C and D denote significant differences (P < 0.05) within a diet over time (comparison across columns). Values that do not share a superscript are significantly different. The detection limit for *Clostridium Cluster* I*, C. perfringens* α toxin I, LT, STa, STb, *E. coli*, K99, F41, F18, F6 and K88 fimbriae was 4 log10 gene copies/g of feces (wet weight).

Figure S1. Fecal microbiota (left) and *Lactobacillaceae* (right) community structure as determined by Principle coordinates analysis (PCoA) of partial 16S rRNA sequences. Each dot represents individual fecal samples, colored according to the feed phase (left) and according to the wheat content of feed (right).

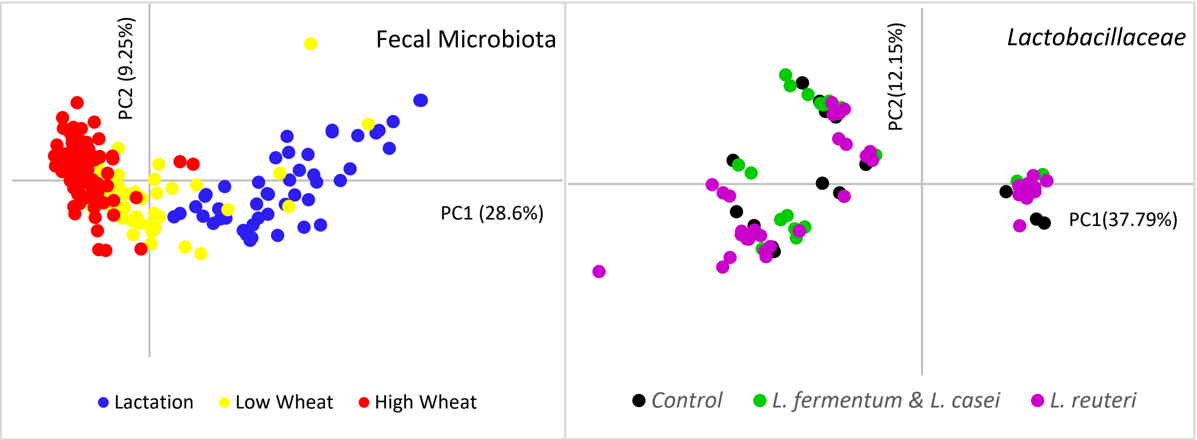

Supplement: Supplementary file 1 [file Data_Sheet_1.PDF]
